# ARP2/3-dependent growth in the plant kingdom: SCARs for life

**DOI:** 10.3389/fpls.2013.00166

**Published:** 2013-06-21

**Authors:** Makoto Yanagisawa, Chunhua Zhang, Daniel B. Szymanski

**Affiliations:** ^1^Department of Agronomy, Purdue UniversityWest Lafayette, IN, USA; ^2^Department of Biological Sciences, Purdue UniversityWest Lafayette, IN, USA; ^3^Department of Botany and Plant Sciences, University of CaliforniaRiverside, CA, USA

**Keywords:** SCAR, WAVE, ARP2/3, actin, ROP, *Arabidopsis*

## Abstract

In the human experience SCARs (suppressor of cAMP receptors) are permanent reminders of past events, not always based on bad decisions, but always those in which an interplay of opposing forces leaves behind a clear record in the form of some permanent watery mark. During plant morphogenesis, SCARs are important proteins that reflect an unusual evolutionary outcome, in which the plant kingdom relies heavily on this single class of actin-related protein (ARP) 2/3 complex activator to dictate the time and place of actin filament nucleation. This unusually simple arrangement may serve as a permanent reminder that cell shape control in plants is fundamentally different from that of crawling cells in mammals that use the power of actin polymerization to define and maintain cell shape. In plant cells, actin filaments indirectly affect cell shape by determining the transport properties of organelles and cargo molecules that modulate the mechanical properties of the wall. It is becoming increasingly clear that polarized bundles of actin filaments operate at whole cell spatial scales to organize the cytoplasm and dictate the patterns of long-distance intracellular transport and secretion. The number of actin-binding proteins and actin filament nucleators that are known to participate in the process of actin network formation are rapidly increasing. In plants, formins and ARP2/3 are two important actin filament nucleators. This review will focus on ARP2/3, and the apparent reliance of most plant species on the SCAR/WAVE (WASP family verprolin homologous) regulatory complex as the sole pathway for ARP2/3 activation.

## INTRODUCTION

Actin filaments are polar, unstable polymers composed of G-actin subunits that reversibly associate with one another through non-covalent interactions. Plant cells contain a population of very short-lived individual actin filaments that polymerize rapidly and are rapidly severed and depolymerized (Staiger et al., 2009; Smertenko et al., 2010; Henty et al., 2011). Direct visualization of the actin network using live cell imaging and variable angle epifluorescence microscopy (VAEM; Konopka and Bednarek, 2008) reveals highly dynamic biochemical activities that enable the network to rearrange in response to cellular needs. The functional role of these plasma membrane-associated unstable filaments in slow growing plant cells with growth rates of 1–10%/h is not known. Actin turnover at the plasma membrane may provide the cell with a mechanism to rapidly monitor cortical domains as part of a “pathogen surveillance” mechanism (Staiger et al., 2009) and/or regulate the activity of K+ channels or other integral membrane proteins at the plasma membrane that participate in turgor regulation and growth control (Liu and Luan, 1998). However, these proposed functional roles are pure speculation.

Part of the difficulty in unraveling the complexity of actin-based functions in plant cells is due to the lack of obvious correlations between the presence of actin filament networks and the growth behavior of plant cells. For example, plant cells that employ an intercalary or diffuse growth mechanism grow slowly, with growth rates of a few %/h, and have highly unstable actin networks that rearrange on the time scales of seconds. Furthermore, beyond a general correlation of longitudinal actin bundles in elongated cells, there are no obvious relationships between specific actin structures and changes in cell shape or plasma membrane deformation. This is in contrast to the situation in non-plant systems in which cortical endocytic actin patches (Evangelista et al., 2002; Kaksonen et al., 2003) and the leading edge of crawling cells (Pollard and Borisy, 2003) define subcellular locations where actin does work to locally control membrane dynamics. In thick-walled plants cells, the magnitude of turgor forces that are required to drive cell expansion exceeds, by orders of magnitude, those that could be generated directly by populations of actin filaments that push on the plasma membrane during the process of polymerization (Szymanski and Cosgrove, 2009). Localized cell wall loosening or the assembly of an anisotropic cell wall generates asymmetric yielding responses to turgor-induced cell wall stress (Baskin, 2005; Cosgrove, 2005). Therefore, actin-based control cell shape is indirect, and the actin cytoskeleton influences cell shape change, in part, by actin and/or myosin-dependent trafficking of cargos, including those that control the localized delivery of protein complexes and polysaccharides that pattern the cell wall (Leucci et al., 2007; Gutierrez et al., 2009). In this scheme for actin-based growth control, the actin network dynamically rearranges within the cell to locally position organelles (Cleary, 1995; Gibbon et al., 1999; Szymanski et al., 1999) and to generate organized roadways that define the patterns of intracellular transport at the whole cell spatial scale (Gutierrez et al., 2009; Dyachok et al., 2011).

It has long been known that acto-myosin transport drives long-distance intracellular transport in plant cells, and motor proteins of the myosin XI class track processively toward actin filament plus ends (Tominaga et al., 2003), to deliver cargo organelles or proteins to specific cellular locations. It is likely that all of the plant myosins are plus-end directed, because the plant myosin VIII and XI motors do not have the insertions, within and adjacent to the motor domain, that are thought to cause the unconventional myosin VI to track toward minus ends of actin filaments (Wells et al., 1999; Menetrey et al., 2005). The polarity of actin filaments within actin bundles and the positioning of actin filaments in the cell are therefore likely to be important because the extent to which filament bundles are aligned within a bundle and the positioning of bundles would determine the efficiency with which myosin motors transport cargos to particular locations in the cell (Szymanski and Cosgrove, 2009). For example, Golgi movement requires myosin activity (Boevink et al., 1998; Avisar et al., 2008, 2009; Peremyslov et al., 2008), and there are strong correlations between the location of actin bundles and the motility patterns of the Golgi (Nebenfuhr et al., 1999; Akkerman et al., 2011). Actin bundles are persistent tracks for a variety of organelles, including the endoplasmic reticulum (ER; Sparkes et al., 2009), and in the shoot of young seedlings, most of membrane-associated myosin localizes to a highly motile ER surface (Ueda et al., 2010). It is therefore possible that organelles that are physically associated with the ER, such as the Golgi and perhaps the *trans*-Golgi network, move by catching a ride with motile ER, and the arrangement of actin bundles in the cell biases the positioning of the secretory pathway to support polarized growth.

This “bundle composition and positioning” control module undoubtedly does not capture the full array of actin-polymerization-dependent activities in the cell, but an importance for bundle composition and positioning in cell shape control is supported by the common observation that actin bundle roadways are not randomly positioned in the cell. In polarized cell types such as leaf trichomes (Szymanski et al., 1999; Le et al., 2003; Deeks et al., 2004; Djakovic et al., 2006) and cylindrical epidermal cells in the root and shoot (Dong et al., 2001; Mathur et al., 2003a; Rahman et al., 2007; Dyachok et al., 2011), the longitudinal alignment of the bundles mirrors the elongated shape of the cells. These bundles tend to be more long-lived structures (Staiger et al., 2009). In addition to defining the general distribution and recycling patterns of organelles in a cell, actin bundles may coordinate the transport of specific cargos. For example, actin bundles and cortical microtubules coordinate the trafficking and insertion of cellulose synthase (CESA)-positive Golgi bodies (Gutierrez et al., 2009), which could allow the cell to maintain the anisotropy of the wall by defining the regions of the cortex with transverse microtubules that receive a continuous supply of CESA, and synthesize cellulose microfibrils with a net transverse alignment.

A major challenge in the field is to determine more broadly the architecture of functionally distinct actin arrays and to discover how the cell uses a diverse collection of actin-binding proteins and filament nucleators to generate these cytoskeletal arrays (Blanchoin et al., 2010). Progress toward this goal is rapidly accelerating: forward and reverse genetic approaches, sophisticated biochemical assays, and live cell imaging are rapidly generating new knowledge about how cytoskeletal proteins orchestrate plant cell responses to endogenous cues and biotic stress, and these topics have been reviewed previously (Smith and Oppenheimer, 2005; Hussey et al., 2006; Yalovsky et al., 2008). This article will focus on a single actin filament-nucleating machine, the actin-related protein (ARP) 2/3 complex, and its relatively simple control in the plant kingdom: positive regulation by a single nucleation promoting factor (NPF) SCAR (suppressor of cAMP receptor) and the WAVE (WASP family verprolin homologous)/SCAR regulatory complex (W/SRC).

## ARP2/3 AND THE NEED FOR PROTEINS THAT PROMOTE ACTIN POLYMERIZATION

The rapid initiation and elongation of actin filaments that is observed *in vivo* is not an intrinsic property of G-actin polymerization. In reactions containing only purified G-actin, the initiation of filament polymerization is slow because trimers of G-actin, which are the seeds for actin polymerization, are rate limiting for the reaction. Eukaryotic cells have evolved an elaborate collection of actin filament nucleators that greatly accelerate filament formation. In plants, the two major known nucleators are the formins (Michelot et al., 2005) and the ARP2/3 complex (Mathur et al., 2003a; Kotchoni et al., 2009). The formin proteins have been reviewed previously (Deeks et al., 2002; Blanchoin and Staiger, 2008), and are a subject of active research (Van Gisbergen et al., 2012). ARP2/3 is an evolutionarily conserved, seven-subunit complex consisting of the actin-related proteins ARP2 and ARP3, and five other distinct subunits, that were originally discovered in *Acanthamoeba* (Machesky et al., 1994). The ARP2/3 complex can promote actin filament nucleation when it is converted from an inactive “open” conformation to a “closed” active conformation in which the actin-related subunits, ARP2 and ARP3, can form a surface that mimics a stable actin dimer and promotes filament nucleation (Robinson et al., 2001; Rodal et al., 2005). ARP2/3 also has F-actin-binding activity, and nucleates actin filaments from the sides of an existing “mother” actin filament at a characteristic 70° angle (Blanchoin et al., 2000). The integration of ARP2/3 into branched actin filament networks may facilitate the physical anchoring of a branched actin network so that the ends of polymerizing actin filaments can do work on an organelle surface.

In living plant cells, roughly one third of the observed filament nucleation events originate from an existing filament or bundle (Staiger et al., 2009); however, it is not yet known if ARP2/3 is responsible for this activity. In non-plant systems, ARP2/3-generated actin filaments do work and function at many different organelle surfaces to deform membranes. Examples include the generation of dendritic actin networks that either drive or consolidate cell shape change in the lamellipodia of some crawling cell types (Svitkina and Borisy, 1999), the promotion of vesicle scission of tubulated membranes that are associated with the late steps of endocytosis (Kaksonen et al., 2005), and cargo sorting activities of early endosomes (Derivery et al., 2009b; Gomez and Billadeau, 2009). However, the precise function of ARP2/3 in plant cells remains enigmatic. Recently, ARP2/3-specific small molecule inhibitors have been identified (Nolen et al., 2009) and have successfully used to *in vivo* analyses of ARP2/3 function in a variety animal cells (reviewed in Rotty et al., 2013). If these inhibitors specifically block ARP2/3 function in plant cells, they could facilitate functional analyses of ARP2/3, especially in species in which ARP2/3 mutants are not available.

Based on mutant phenotypes and gene expression patterns, ARP2/3 and the W/SRC participate widely in plant growth and development. W/SRC and ARP2/3 mutants in the moss *Physcomitrella patens *(Harries et al., 2005; Perroud and Quatrano, 2006), *Arabidopsis *(Le et al., 2003; Li et al., 2003; Mathur et al., 2003a; Brembu et al., 2004; Deeks et al., 2004), and maize (Frank and Smith, 2002) have demonstrated the broad importance of ARP2/3 and its activation during cell morphogenesis. Tip growing cells have a unique actin-dependent cytoplasmic organization that defines the streaming patterns of the cell and dictates the mechanical properties of the apex by restricting vesicle exchange with the plasma membrane to this region of the tip growing cell (Daher and Geitmann, 2011; Palin and Geitmann, 2012). In moss, ARP2/3 is a critical component of the tip growth machinery, because mutant protonemal cells grow very slowly and with an altered geometry compared to the wild type (Harries et al., 2005; Perroud and Quatrano, 2008). However, ARP2/3 is not universally required during tip growth in higher plants. This is based on the normal transmission of null ARP2/3 alleles through pollen, and the lack of a clear root hair phenotype in *Arabidopsis *(Le et al., 2003; Djakovic et al., 2006). However, based on reports from two model species that are used for nodulation research, ARP2/3 clearly has a function in some aspects of root hair development because mutation of genes in the ARP2/3 pathway caused a failure of root hairs to develop functional infection threads and normal root nodules (Yokota et al., 2009; Miyahara et al., 2010). An importance for ARP2/3 during cell morphogenesis is more obvious in cells that employ a diffuse growth mechanism. Based on mutant phenotypes in maize and *Arabidopsis*, the size and shapes of epidermal cells throughout the entire plant are affected (Frank and Smith, 2002; Le et al., 2003; Mathur et al., 2003a). In the distorted class of *Arabidopsis* ARP2/3 mutants, leaf trichomes are obviously swollen and twisted due to the constrained importance of an organized actin cytoskeleton to maintain polarized growth in this cell type (Szymanski et al., 1999). The distorted mutants and *Arabidopsis spike1 *(*spk1*) may also affect the physical properties of the middle lamella and cell–cell adhesion (Qiu et al., 2002). For example, *spk1 *and the distorted mutants differ from most other morphology mutants in that they display gaps in the shoot epidermis, most frequently at the interface of pavement cells and stomata (Qiu et al., 2002; Le et al., 2003; Mathur et al., 2003a; Zhang et al., 2005; Djakovic et al., 2006). The cell gaps may reflect defective cortical actin-dependent secretion of polysaccharides and/or proteins that promote cell–cell adhesion (Smith and Oppenheimer, 2005; Hussey et al., 2006; Leucci et al., 2007). However, the endogenous cargo that is putatively affected in the distorted mutants is not known, and the possible contribution of mis-regulated cell expansion between adjacent cells needs to be examined further. In the distorted mutants, the relative amount of actin filaments are slightly reduced compared to the wild type (Le et al., 2003) and the distorted phenotypes are relatively mild, suggesting that other nucleators, such as formins generate the bulk of the actin network. In budding yeast, ARP2/3 and formins have largely independent functions, and nucleate endocytic patches and actin cables that facilitate cell polarization, respectively (Evangelista et al., 2002). On the other hand, ARP2/3 and the formin FMNL2 cooperate in lamellipodia to generate fully functional dendritic actin networks (Block et al., 2012). It remains to be determined how plant cells deploy these different classes of nucleators during morphogenesis.

## A SIMPLIFIED SCHEME FOR ARP2/3 ACTIVATION IN PLANTS; ONLY WAVE/SCAR

A diverse class of proteins termed NPFs converts inactive ARP2/3 into a potent actin filament-nucleating machine (reviewed in Welch and Mullins, 2002; Stradal and Scita, 2006). For example, WASP (Wiskott-Aldrich syndrome protein)-family proteins were the first endogenous NPFs to be discovered (Rohatgi et al., 1999; Winter et al., 1999; Yarar et al., 1999). WASP-family proteins have a conserved C-terminal WA domain sequential G-actin-binding WASP-homology 2 (WH2) and a signature acidic motif that was shown to be necessary and sufficient for ARP2/3 activation. The same domain was subsequently identified in another ARP2/3 activator termed WAVE (Miki et al., 1998b), and the WA domain continues to provide a way to identify additional ARP2/3 activators such as WHAMM, JMY, and the WAVE/SCAR homologous protein WASH (see the excellent review by Rottner et al., 2010). NPFs are effectors that convert small GTPase and/or lipid signals into spatially and temporally defined ARP2/3 activation responses. NPFs may also employ specific phospholipid- (Rohatgi et al., 2000; Oikawa et al., 2004) or F-actin- (Goode et al., 2001) binding activities to locally concentrate ARP2/3 either on the surface of particular organelles or within particular actin networks, respectively. It is now apparent that metazoan cells use an assortment of heteromeric complexes to couple signal transduction cascades to actin dynamics and multiple organelle surfaces (Stradal and Scita, 2006; Rottner et al., 2010). Current research efforts are focused on identifying the components and cellular control mechanisms of NPF-based signaling and determining exactly how localized ARP2/3 activation within subdomains of organelles affects the dynamics of endogenous proteins and/or lipids.

**FIGURE 1:**
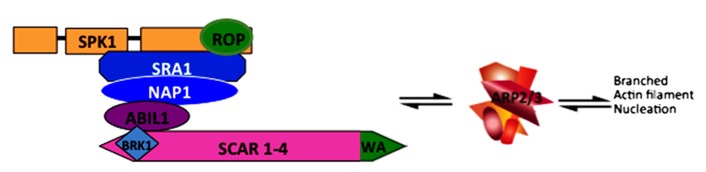
**Depiction of theW/SRC and ARP2/3 complexes leading to an actin filament nucleation response.** W/SRC is positively regulated by the DOCK-family GEF SPK1 and ROP-GTP. In the fully activated state the WA domain (colored green) of SCAR proteins physically interacts with and activates ARP2/3. The output of the pathway is the generation of branched actin filaments in which ARP2/3 binds to the side of a mother filament and nucleates a new filament.

In the plant kingdom ARP2/3 activation is highly simplified and appears to rely solely on the WAVE/SCAR family of NPFs (hereafter termed SCAR) and the heteromeric WAVE/SCAR regulatory complex (hereafter termed W/SRC; Eden et al., 2002). A cartoon depicting the subunit composition and overall regulatory relationships between W/SRC, ARP2/3, and actin filaments is shown in **Figure 1**. Evidence for the singular importance of SCAR for ARP2/3 activation in plants is based primarily on genetic data from *Arabidopsis*: lines that are null for *ARP2/3*, *SCAR*, and single copy W/SRC subunit genes have an identical array of equally severe phenotypes (Szymanski, 2005). This result does not exclude the possibility of SCAR-independent pathways in *Arabidopsis *because not all ARP2/3 phenotypes are known. Furthermore, there is some evidence for additional ARP2/3 activators in the plant kingdom. For example, although searches for WA domain-containing proteins did not reveal additional candidates in *Arabidopsis *or obvious homologs of WASP, WHAMM, or JMY (D.S., unpublished), two publications centered on the sequence analyses of WASP-family protein evolution report the presence of WASH-homologous proteins in a small number of plant species(Veltman and Insall, 2010; Kollmar et al., 2012). The WASH regulatory complex is an interesting case because each of its subunits is structurally similar to the analogous W/SRC subunit, but the structurally similar protein pairs are so diverged at the sequence level, that sequence comparisons cannot identify any similarity (Jia et al., 2010). With the exception of the green algae *Ostreococcus lucimarinus*, which appears to encode a homolog of each of the five WASH regulatory complex subunits including a WASH subunit with an intact WA domain, no other plant species has been reported to encode the full set of WASH regulatory complex subunits (Kollmar et al., 2012). In most species with sequenced genomes, WASH complex genes are either absent or exist as fragmented pseudogenes that are unlikely to have a functional importance. If these genome-sequence-based protein sequence predictions are correct, the data indicate that WASH complex genes were present in a common ancestor of plants and metazoans, but loss of WASH genes and retention of W/SRC has occurred widely within the plant kingdom.

## W/SRC: A SINGULAR RELIANCE ON SCAR IN MOST PLANT SPECIES

In plants, unlike in other systems (Ibarra et al., 2006; Weiner et al., 2006; Anitei et al., 2010), there is no evidence to date, that W/SRC subunits have any functions independent of the SCAR subunit and an ARP2/3 activation pathway. W/SRC regulation is therefore likely to be relatively simple compared to other systems, and more experimentally tractable. The existence of *trans*-regulating factors for SCAR/WAVE was originally hypothesized because the full-length proteins (Machesky et al., 1999), unlike the autoinhibited NPF N-WASP (Miki et al., 1998a), are intrinsically active and potentially disruptive to the cell if not properly regulated. A biochemical search for *trans*-regulating factors led to the original identification of the five subunits of the W/SRC (Eden et al., 2002). Shortly thereafter, forward genetic screens in *Arabidopsis* based on a distorted trichome phenotype identified homologs of both the W/SRC and ARP2/3 complex subunits (**Figure 1**).

**FIGURE 2:**
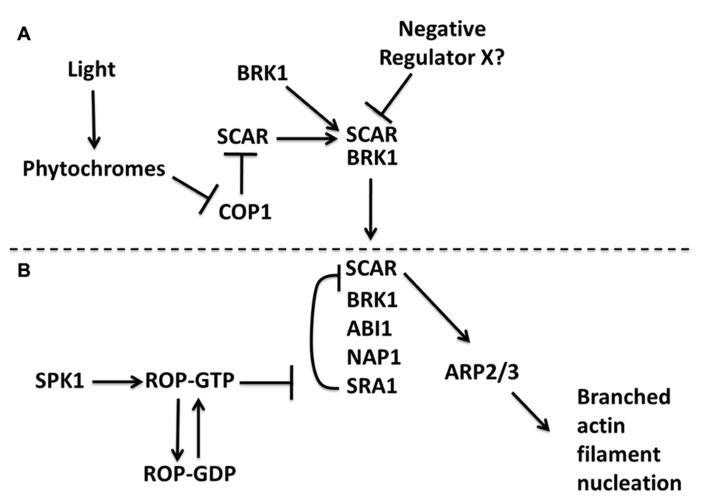
**A model for the genetic and biochemical control of SCAR and W/SRC in *Arabidopsis*.** Regulation of SCAR protein stability is proposed to occur independent of fully assembled W/SRC **(A)**. A light signal leads to phytochrome-dependent stabilization of SCAR protein. A physical association of SCAR with BRK1 also stabilizes SCAR. An unknown component is proposed to negatively regulate the BRK1–SCAR sub-complex. Regulation of the fully assembled W/SRC that forms when the BRK1–SCAR sub-complex associates with a proposed NAP1–SRA1–ABI sub-complex **(B)**. SPK1 generates activating ROP-GTP signals that antagonize SRA1-dependent *trans*-inhibition of SCAR. The output of the pathway is presumed to be the generation of a network of branched actin filaments. The dashed line emphasizes the distinct levels of control between partial and fully assembled W/SRC.

In general, the signaling proteins and regulatory schemes that control NPF activity are not well understood. In the case of the W/SRC, it is a known ROP (Rho-of-Plants)/Rac effector complex that converts ROP/Rac-GTP and other signals into a localized ARP2/3 activation response (Miki et al., 1998b; Basu et al., 2004; Uhrig et al., 2007). The exact nature of the cellular control of the W/SRC–ARP2/3 pathway is complex and poorly understood (see also below); however, a direct physical interaction between active ROP/Rac proteins and the W/SRC subunit SRA1 appears to be evolutionarily conserved and an important aspect of pathway control (Basu et al., 2004; Lebensohn and Kirschner, 2009; Chen et al., 2010). There was an initial period of controversy concerning a proposed mechanism of Rac-dependent W/SRC disassembly and loss of WAVE/SCAR *trans*-inhibition (Eden et al., 2002). However, it is now apparent that vertebrate W/SRC is intrinsically inactive (Derivery et al., 2009a; Ismail et al., 2009), and the genetic data in *Arabidopsis* are completely consistent with the plant W/SRC being intrinsically inactive (Szymanski, 2005). Ligands such as Rac (Chen et al., 2010), and perhaps specific phospholipids (Lebensohn and Kirschner, 2009), cause conformational changes in vertebrate W/SRC that allow it to activateARP2/3. A recent crystallographic analysis of the vertebrate W/SRC reveals a plausible mechanism for ROP/Rac positive regulation of W/SRC, in which the active forms of the small GTPase and the WA domain of SCAR bind to the same region of the SRA1 subunit (Chen et al., 2010). Active ROP/Rac proteins may therefore antagonize *trans*-inhibition of the SCAR subunit that is assembled into W/SRC, and promote conformational changes in the complex that can lead to ARP2/3 activation (**Figure 2B**). It is important to add that high concentrations of Rac are required to fully activate W/SRC* in vitro*, and additional ligands (Lebensohn and Kirschner, 2009; Koronakis et al., 2011) and protein phosphorylation control (Ura et al., 2012) may also be required to fully activate W/SRC in cells. The function of the W/SRC is therefore to integrate multiple signaling inputs to determine where and when the SCAR subunit activates ARP2/3.

## SCAR: MULTIPLE DOMAINS MEDIATING W/SRC ASSEMBLY AND ARP2/3 ACTIVATION

**FIGURE 3:**
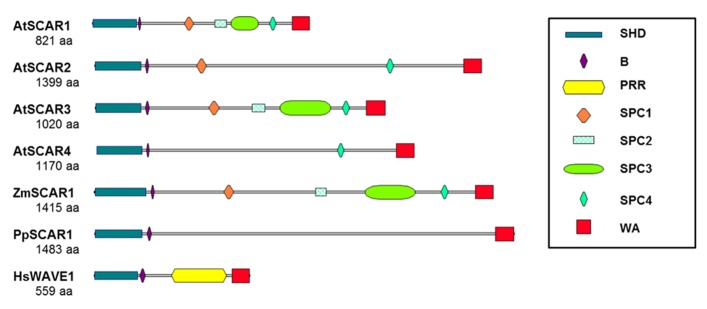
**The domain organization of plant SCARs.** Functional SCARs are defined as proteins containing conserved N-terminal SHD and C-terminal WA domains. Plant SCARs differ from those of other organisms, including human, in that they do not have PRR. The large central region of plant SCARs, instead, contains one or more plant-specific conserved motifs (SPC1 through 4), except PpSCAR1. Additional conserved regions within the central domain of SCARs are labeled as defined in the legend to the figure. SCAR sequences are from *Arabidopsis* (AtSCAR1: NP_001031474, AtSCAR2: NP_181378, AtSCAR3: NP_001117376, and AtSCAR4: NP_195793), maize (ZmSCAR1: AFW80370), moss (PpSCAR1: XP_001754078) and human (HsWAVE1: NP_003922). SHD, SCAR homology domain; B, basic region; PRR, proline-rich region; SPC, SCAR of plant central region; WA, WH2 (W), central, and acidic (A) domains.

The complexity of SCAR regulation is reflected in the presence of multiple conserved domains with distinct functional roles. The SCAR WA domain is the best characterized and contains highly conserved elements (Panchal et al., 2003) that are preserved throughout evolution as hallmarks of ARP2/3 activators (Basu et al., 2005). The WA domain binds to both G-actin and the ARP2/3 complex (Marchand et al., 2001), and both activities are detected with plant SCARs (Deeks et al., 2004; Frank et al., 2004; Basu et al., 2005; Uhrig et al., 2007). In fact, plant SCARs were first identified based on the presence of a WA domain in several maize and *Arabidopsis* proteins and the ability of purified WA fragments to activate vertebrate ARP2/3 (Frank et al., 2004). All plant SCARs contain an intact WA domain (**Figure 3**); however, they differ in the efficiency with which they activate ARP2/3. For example, in one comparative study *Zea mays* SCAR1 potently activated ARP2/3, whereas *Arabidopsis* SCAR4 had limited activity (Frank et al., 2004). Based on a genetic and biochemical study of SCARs in *Arabidopsis*, the efficiency with which different SCARs activate ARP2/3 is correlated with the relative importance of individual SCAR genes *in vivo* (Zhang et al., 2008).

Plant SCARs are usually encoded by small gene families, unlike many of the other W/SRC and ARP2/3 subunit genes, which are often single copy. For example, *Arabidopsis* encodes four functional SCAR isoforms, and *DISTORTED3*/*IRREGULAR TRICHOME BRANCH 1/SCAR2* was the first to be identified genetically because mutations in *SCAR2 *were sufficient to cause a mild distorted trichome phenotype (Basu et al., 2005; Zhang et al., 2005). The overall domain organization of SCAR2 is similar to other SCARs present in lower plants such as the moss *P. patens *and grass species such as maize (**Figure 3**). All plant SCARs have a C-terminal WA domain and a conserved N-terminal SHD (SCAR homology domain; **Figure 3**). SHD is involved in W/SRC assembly, and mediates direct physical interactions with the W/SRC subunits ABIL1 (ABL interactor like protein 1; Basu et al., 2005) and BRK1 (Frank et al., 2004). The physical interaction of SHD with BRK1 is highly significant, because BRK1 specifically stabilizes SCAR proteins (Djakovic et al., 2006; Le et al., 2006). Plant SCARs also contain a cluster of basic amino acids just C-terminal to the SHD (**Figure 3**). In non-plant systems, the basic region has been proposed to determine the localization of SCARs by binding to PtdIns (3,4,5) P_3_ (Oikawa et al., 2004) and to mediate auto-inhibitory ionic interactions with the WA domain that occur when WA is highly phosphorylated (Ura et al., 2012). However, the importance of the basic region in plant SCARs is not known. The distinguishing feature of plant SCARs is the presence of a large and highly variable central region (**Figure 3**). Unlike the central regions of vertebrate WAVE, which is dominated by a proline rich region (**Figure 3**), that binds to both the G-actin-binding protein profilin and Src homology 3-domain-containing signaling proteins (Miki et al., 1998b), plant SCARs lack a large proline rich region; however, they do contain a short conserved proline rich motif at the near C-terminus that may have a functional importance. A comprehensive bioinformatics analysis of the WAVE/SCAR family proteins identified several conserved sequence features within the plant SCAR central region that merit further study (Kollmar et al., 2012). We used the sequences by Kollmar et al. (2012) (**Table 1**) to map the motifs onto a selected set of known plant SCAR proteins. In order to create a consistent nomenclature, these regions of conserved sequences are referred to as SCAR of plants central region (SPC) 1 through 4. The conserved regions are numbered according to their order of occurrence within AtSCAR1, AtSCAR3, and ZmSCAR1. The restricted occurrences of SPC2 and SPC3 within a subset of plant SCARs is consistent with previous phylogenetic analyses that defined two clades of plant SCARs based on an analysis of the SHD and WA domains (Zhang et al., 2008). The SPCs are not detected in the moss protein PpSCAR1, suggesting that these motifs evolved to carry out functions that are unique to higher plants.

**Table 1:** Amino acid sequences used for motif search in plant SCARs.

SPC1	SDGSHSDDIESEVDNYMDALNTMESESETDNECQTKR
(=WAM1*)		
SPC2	SSSCESQESLAESSSVHSVKFWTNGGLLGLEPSKPPDFAVSNSL
(=WAM3)		
SPC3	GLGHRLLINGFQRKVSLVHDDLKMEPASSLKSGALEQESGHNSVGY
(=WAM4)	QAEPETTFKEQFGNKTENGMDGLSKSSIFGSPIDSLPSSPPLEHMK
	ISFHPIDGFETSKLKLKFPDGNHHESVRDMFPSFQLVPEPSIPLHDSG
	SDSDDDDTFCRSSPYMSDDCLSHHSESNSSEQWESD
SPC4	EMPPPPPLPPMQWRLGKPQLGSLEEK
(=WAM2)		

* Nomenclature used by Kollmar et al. (2012) is shown in parentheses.

## THE GENETICS AND CELL BIOLOGY OF SCAR IN *Arabidopsis*

Thorough genetic analyses of the SCAR gene family showed that, despite the clear differences in amino acid sequence among the SCARs, the proteins are functionally interchangeable in the context of ARP2/3-dependent cell shape control. For example, functional redundancy among SCARs was the likely explanation of the relatively weak trichome phenotype of *scar2 *(Basu et al., 2005). Subsequent analyses of the *Arabidopsis SCAR *gene family revealed surprising and informative regulatory relationships. In one paper, it was shown that although *scar4* trichomes had no phenotype, *scar2*;*scar4* double mutants had severely distorted trichomes that were more severe than *scar2* alone and similar to those of null *arp2/3* mutants (Uhrig et al., 2007). The cell type-specific phenotypes were hypothesized to be due to isoform-specific functions of SCARs and the assembly of functionally distinct types of W/SRCs. Subsequently, a comprehensive genetic analysis of the *SCAR* gene family and ectopic expression of a SCAR3 showed that, although cell types differed with respect to the relative importance of different *SCAR*s, the genes can function interchangeably (Zhang et al., 2008). The importance of individual SCARs within a cell type correlated with gene expression levels and with the biochemical efficiency with which an individual isoform would activate ARP2/3. These data are the basis for a SCAR activity threshold model (Zhang et al., 2008), in which cell types have differing requirements for ARP2/3 activation, and any combination of SCARs can be used to adequately activate ARP2/3. The cellular explanations of cell type-specific differences for ARP2/3 activation remain to be determined.

SCARs are subjected to multiple levels of post-translational control. One is at the level of protein stability. In most non-plant systems all W/SRC subunits have a mutually dependent protein stability, and removal of any one subunit strongly decreases the protein levels of all of the other subunits (Derivery and Gautreau, 2010). This is not the case in *Arabidopsis*, as loss of BRK1/HSPC300 specifically destabilizes SCAR2, but not the W/SRC subunit NAP1 (nucleosome assembly protein 1; Le et al., 2006). In contrast, SCAR2 is not degraded in the *nap1* or *sra1* null backgrounds. SCAR2 is not ectopically active in *nap1* and *sra1*, because these mutants have loss of function cell shape and actin cytoskeleton phenotypes (Basu et al., 2004; Brembu et al., 2004; Deeks et al., 2004; El-Assal et al., 2004; Li et al., 2004). This indicates that the pool of SCAR in the mutants has little or no biological activity, and stable SCAR or BRK1–SCAR sub-complexes in *nap1* and s*ra1* are subject to additional levels of negative regulation (**Figure 2A**). The response of SCAR to loss of SRA1 in *Arabidopsis *differs from that of the amoebae *Dictyostelium*. In this organism, SCAR levels are greatly reduced in *sra1*, but the residual SCAR is hyperactive, ectopically activating ARP2/3 actin meshwork formation (Blagg et al., 2003). In plants, W/SRC assembly may be an important point of control in terms of defining the cellular levels of fully assembled complex. It is also possible that reversible W/SRC assembly could play an important regulatory role as W/SRC is cycled through multiple rounds of activation, use, and inactivation. Although the concept of reversible W/SRC assembly is controversial (Bompard and Caron, 2004), the potential regulatory importance of complex assembly deserves further study (Derivery and Gautreau, 2010).

BRK1 is an interesting ~8 kDa evolutionarily conserved W/SRC subunit that may be involved in determining the cellular levels of SCAR and the extent to which it is fully assembled into the W/SRC (Derivery et al., 2008). Plant BRK1 was first cloned in maize based on the reduced crenulation of epidermal cells of the *brick1* mutant (Frank and Smith, 2002). It was subsequently shown that BRK1 physically interacts with the conserved N-terminal SHD of SCAR (Frank and Smith, 2002). The idea that BRK1 masks a structural feature in the SCAR SHD that otherwise promotes proteasome-dependent protein turnover remains untested (Le et al., 2006), and in our hands proteasome inhibitors do not rescue *brk1 *shoot phenotypes (D.S., unpublished). However, it was recently shown that the proteasome inhibitor MG132 can stabilize a nuclear localized pool of SCAR, and constitutive photomorphogenic 1(COP1)- and proteasome-dependent turnover of SCAR in root cells is associated with reduced elongation in etiolated seedlings (Dyachok et al., 2011). It is possible that BRK1 and proteolytic pathways operate antagonistically to define the cellular concentration of BRK1–SCAR sub-complexes that may participate in W/SRC assembly and ARP2/3 activation (**Figure 2**).

Most research has focused on fully assembled W/SRC, which is widely regarded to be the entity that converts cellular signals into an ARP2/3 activation response (Steffen et al., 2004; Chen et al., 2010). The crystal structure for vertebrate W/SRC was recently solved and provided insight into the mechanism of *trans*-inhibition, in which the SRA1 subunit of W/SRC physically interacts with the SCAR-WA domain, and sterically inhibits a physical interaction with ARP2/3. GTP-bound Rac was shown to bind to the same region of SRA1 and provides a plausible mechanism for activation via relief of *trans*-inhibition. However, the nature of W/SRC positive regulation remains poorly understood because high concentrations of Rac-GTP are required for activation, and in other systems multiple sources for W/SRC positive regulation have been identified (Ismail et al., 2009; Lebensohn and Kirschner, 2009; Koronakis et al., 2011; Ura et al., 2012).

The vertebrate W/SRC structure and its regulation are relevant to plants, because the subunits, complex assembly mechanisms, and regulatory schemes of W/SRC–ARP2/3 pathway in *Arabidopsis* and *Physcomitrella* are conserved compared with vertebrate species (Szymanski, 2005). For example, plant genomes encode subunits of each of the W/SRC subunits, and transgene-rescue experiments in *Arabidopsis* indicate that human W/SRC complex subunits can substitute for the *Arabidopsis* proteins (Basu et al., 2004; El-Assal et al., 2004). Furthermore, biochemical assays of *Arabidopsis* W/SRC have shown that the binary interactions among W/SRC subunits that mediate complex assembly are indistinguishable from those that have been observed for human W/SRC (Gautreau et al., 2004) and are mediated by conserved domains within the individual subunits (Basu et al., 2004; El-Assal et al., 2004; Frank et al., 2004; Le et al., 2006; Uhrig et al., 2007).

The mechanism by which active ROP is coupled to W/SRC is being resolved. In non-plant systems the identity of the GEF (guanine nucleotide exchange factor) that transmits activating Rac signals to W/SRC is not known. In *Arabidopsis*, the DOCK (dedicator of cytokinesis)-family GEF SPK1 functions as an upstream activator of ROP and W/SRC based on genetic epistasis tests and a physical association of endogenous SPK1 with W/SRC complex subunits (Basu et al., 2008). This conclusion is further supported by the detection of a yeast 2-hybrid interaction between SPK1 and the ABIL W/SRC subunit (Uhrig et al., 2007), which is a known W/SRC subunit (Jorgens et al., 2010) that physically interacts with SCAR (Basu et al., 2005). We propose a model in which SPK1 is a transient component of fully assembled W/SRC complexes that transmits activating ROP-GTP signals that antagonize SRA1-dependent *trans*-inhibition of SCAR (**Figure 2B**). The involvement of ROPs in trichome morphogenesis has been difficult to determine because so many ROPs are expressed in trichomes (Marks et al., 2009), and recessive ROP mutants with clear trichome phenotypes have yet to be reported, even though some have an effect on pavement cell shape (Fu et al., 2005; Xu et al., 2010). A recent report describes a clever strategy to generate ROP loss of function lines that used the ectopic expression of ROP-specific bacterial toxins. There was a strong association between inducible expression of the toxins and the appearance of trichomes with severe trichome swelling and reduced branch number phenotypes (Singh et al., 2012). Given the involvement of ROPs, the identification of a ROPGEF SPK1 (Qiu et al., 2002; Basu et al., 2008), and ROP effectors in the W/SRC–ARP2/3 pathway (Basu et al., 2004; Uhrig et al., 2007), it is now possible to construct detailed biochemical and genetic models for pathway control (**Figure 2**). The major challenge now is centered on learning how plant cells deploy these important growth-controlling machines.

## POTENTIAL MECHANISMS FOR THE CELLULAR CONTROL OF W/SRC AND ARP2/3

Localization data from non-plant systems suggest that W/SRC is partitioned between active and inactive pools. For example, in cultured human cell lines that crawl on a solid substrate, current models propose that a cytosolic pool of inactive SCAR proteins and W/SRC are locally recruited and activated at specific plasma membrane surfaces in response to activating signals (Oikawa et al., 2004; Lebensohn and Kirschner, 2009; Chen et al., 2010). However, in *Drosophila* neurons (Bogdan and Klambt, 2003) and cultured human melanoma cells (Steffen et al., 2004), there are large pools of W/SRC with a perinuclear and organelle-like punctate localization that have no obvious relationship to cell shape or motility, raising uncertainty about the cellular mechanisms of W/SRC activation and the importance of different organelle systems. In plants, cell fractionation experiments indicate that SCAR1 and ARP2/3 have an increased association with membranes compared with their animal counterparts (Dyachok et al., 2008; Kotchoni et al., 2009). In tip growing moss protonemal cells, both BRK1 and ARP2/3 localize to a population of unidentified organelles within the apical zone (Perroud and Quatrano, 2008). Similar live cell imaging experiments in *Arabidopsis* reported a plasma membrane localization for SCAR1 and BRK1 in a variety of shoot epidermal and root cortex, and their accumulation at young trichome branch tips and at 3-way cell wall junctions may define subcellular domains for W/SRC–ARP2/3-dependent actin filament nucleation at the plasma membrane (Dyachok et al., 2008). However, to our knowledge, active W/SRC, defined here as the fraction of W/SRC that colocalizes with ARP2/3 or actin, has not been reported in plants, and the plasma membrane is not necessarily the only organelle involved in W/SRC regulation. For example, the reported accumulation of BRK1 and SCAR1 at 3-way cell wall junctions has a punctate appearance at the cell cortex that may not simply correspond to the plasma membrane (Dyachok et al., 2008). Also, in young stage 4 trichomes, there was an uncharacterized pool of intracellular SCAR1, but not BRK1, that localized to intracellular punctate structures (Dyachok et al., 2008). The ER may also be involved in W/SRC regulation. SPK1, the known upstream regulator W/SRC is localized to specialized domains of the ER termed ER exit sites (Zhang et al., 2010). We recently discovered a large pool of inactive NAP1 and SCAR2 on the ER surface. NAP1 localization in mutant backgrounds and colocalization with actin clearly showed that the ER-localized pool is probably inactive with respect to full W/SRC assembly and ARP2/3 activation (Zhang et al., 2013). These results provide additional support for the importance of the ER surface in the control of W/SRC regulation.

We propose that multiple organelles participate in W/SRC regulation and function, and that full activation and deployment of the complex occurs in multiple stages because the localization patterns of BRK1, SCAR, and NAP1 in identical cell types do not completely overlap (Dyachok et al., 2008, 2011; D.S. and C.Z., unpublished). We also propose that pools of W/SRC sub-complexes exist (**Figure 2**), but it will be important to determine the extent to which the different localization patterns among the subunits are explained by either real differences among the subunits in their assembly status or artifacts due to the particular localization methods and/or the particular fluorescent protein fusions that are used. The ER surface appears to be a general platform that accumulates W/SRC subunits in either an unassembled or partially assembled state. It is possible that full complex assembly and the initial SPK1-dependent loading of ROP into the W/SRC occur at specialized subdomains of the ER that also contain components of COPII-dependent ER-export.

If the ER and ERES (ER exit sites) are sites for W/SRC positive regulation, this would raise many interesting questions regarding the biological rationale for using the ERES as a hub for ROP-dependent signal transduction and actin polymerization. At present the importance of actin filaments associated with ERES is unclear. The vesicle transport from ER to the Golgi does not strictly require an intact actin cytoskeleton (Saint-Jore et al., 2002), and in general there is limited colocalization between the ER and actin networks (Ueda et al., 2010). It may be that ARP2/3 is activated at specific sub-domains of the ER to either polymerize actin filaments that do work and promote localized membrane tubulation or vesicle formation. It is also conceivable that ARP2/3 generates stable ER-associated actin meshworks that could act as a scaffolding element that recruits other actin-binding proteins and motor proteins that control organelle positioning or biogenesis. On the other hand, the ER surface may not be a site for ARP2/3 activation. Perhaps, the ER-associated W/SRC is not fully activated, and there is a spatial uncoupling of early SPK1-dependent positive regulation of W/SRC at the ER surface. The ultimate full activation of W/SRC and ARP2/3 complexes may occur at some other membrane surface. Clearly additional localization and live cell imaging studies are needed to define more precisely the locations of active ARP2/3 and its importance in terms of actin cytoskeleton reorganization and growth control.

## FUTURE PERSPECTIVES

The knowledge base concerning the components and regulation of the plant W/SRC and ARP2/3 pathway is approaching what is needed to predict and manipulate the signaling inputs and actin polymerization outputs of the pathway. Further research efforts in this area could have value, not only as a test case for engineering the plant cytoskeleton, but also as a strategy to improve the value of crop species. W/SRC–ARP2/3 is involved in so many processes that contribute to important crop traits such as stomatal dynamics and water use efficiency (Jiang et al., 2012), infection thread formation during the process of root nodulation (Yokota et al., 2009; Miyahara et al., 2010; Hossain et al., 2012), and cellular growth control that impacts organ architecture and the adhesive properties of cells in the context of a tissue (Mathur et al., 2003b). The current barrier to meeting these lofty goals is a lack of sufficient details concerning the precise cellular function of ARP2/3-generated actin filaments in plant cells.

## Conflict of Interest Statement

The authors declare that the research was conducted in the absence of any commercial or financial relationships that could be construed as a potential conflict of interest.

## Abbreviations

ABIL: ABLinteractor like protein
ADF: ADF, actin-depolymerizing factor
ARP2/3: actin-related protein 2/3 complex
CESA: cellulose synthase
ERES: ER exit sites; F-actin, filamentous actin; G-actin, globular actin
GEF: guanine nucleotide exchange factor
NPF: nucleation promoting factor
ROP: Rho-of-Plants
SCAR: suppressor of cAMP receptor
SHD: SCAR homology domain
WASP: Wiskott-Aldrich syndrome protein
WAVE: WASP family verprolin homologous.
